# Vitamin E supplementation prevents obesogenic diet-induced developmental abnormalities in SR-B1 deficient embryos

**DOI:** 10.3389/fcell.2024.1460697

**Published:** 2024-10-09

**Authors:** Alonso Quiroz, Gabriela Belledonne, Fujiko Saavedra, Javier González, Dolores Busso

**Affiliations:** ^1^ PhD Program in Medical Science, Faculty of Medicine, Pontificia Universidad Católica de Chile, Santiago, Chile; ^2^ Ph.D. Program in Biomedicine, Faculty of Medicine, Universidad de los Andes, Santiago, Chile; ^3^ Faculty of Medicine, Universidad de los Andes, Santiago, Chile; ^4^ Biomedical Research and Innovation Center, Faculty of Medicine, Universidad de los Andes, Santiago, Chile; ^5^ Center of Interventional Medicine for Precision and Advanced Cellular Therapy (IMPACT), Santiago, Chile

**Keywords:** embryo development, neural tube defects, malnutrition by excess, vitamin E, SR-B1 deficiency

## Abstract

**Introduction:**

Genetic and environmental factors influence the risk of neural tube defects (NTD), congenital malformations characterized by abnormal brain and spine formation. Mouse embryos deficient in Scavenger Receptor Class B Type 1 (SR-B1), which is involved in the bidirectional transfer of lipids between lipoproteins and cells, exhibit a high prevalence of exencephaly, preventable by maternal vitamin E supplementation. SR-B1 knock-out (KO) embryos are severely deficient in vitamin E and show elevated reactive oxygen species levels during neurulation.

**Methods:**

We fed SR-B1 heterozygous female mice a high-fat/high-sugar (HFHS) diet and evaluated the vitamin E and oxidative status in dams and embryos from heterozygous intercrosses. We also determined the incidence of NTD.

**Results and discussion:**

HFHS-fed SR-B1 HET females exhibited altered glucose metabolism and excess circulating lipids, along with a higher incidence of embryos with developmental delay and NTD. Vitamin E supplementation partially mitigated HFHS-induced maternal metabolic abnormalities and completely prevented embryonic malformations, likely through indirect mechanisms involving the reduction of oxidative stress and improved lipid handling by the parietal yolk sac.

## 1 Introduction

Neural tube closure defects (NTD) constitute a group of congenital malformations caused by the abnormal formation of the neural tube, an embryonic structure that gives rise to the brain and spine. Among humans, neural tube defects (NTDs) exhibit a global prevalence of approximately 2 cases per 1,000 pregnancies, positioning them as one of the prevalent birth abnormalities (Kancherla, 2023). The etiology of NTD is complex and includes genetic and environmental determinants, including pregestational exposure to toxicants or anticonvulsants and nutritional or metabolic imbalances, i.e., maternal folate deficiency, diabetes and obesity (Greene and Copp, 2014).

A strong indication that an altered maternal metabolism contributes to congenital malformations comes from diabetic embryopathies (Garne et al., 2012). In mothers with pregestational diabetes, the risk of having a child with NTD is up to 4 times higher than in non-diabetic women (Bell et al., 2012). In the last decade, maternal obesity has also been established as an independent risk factor for NTD, with a relative risk of between 2 and 3 reported in women of different ethnicities with obesity vs. women with adequate body mass index (Rasmussen et al., 2008; Gao et al., 2013; Shaw et al., 1996). Malnutrition, defined as consumption of poor-quality diets, particularly those with low provision of micronutrients and high contribution of calories derived from fats and sugars, also increases the risk of NTD (Carmichael et al., 2012; Sotres-Alvarez et al., 2013; Yazdy et al., 2010). The best-described nutritional deficiency associated with an increased NTD risk is folate deficiency (Crider et al., 2022). In pregnancies with abnormal metabolism and nutritional status, consuming high doses of folic acid supplements preconceptionally prevents NTD significantly (Sotres-Alvarez et al., 2013; Petersen et al., 2019; Correa et al., 2012). However, the existence of folic-acid-resistant cases justifies further research to design new preventive strategies for NTD, particularly in high-risk groups.

Due to the inaccessibility of the human embryo during the neurulation stage, studying neurulation in mice has enabled to gain knowledge on the mechanisms elucidating NTD (Harris and Juriloff, 2007). Most of the studies have been focused on understanding the role of specific micronutrients during mouse neurulation. Conversely, malnutrition by excess as a potential risk factor for NTD has received comparatively less attention. Two decades ago, Harris and Jurriloff demonstrated in a genetically susceptible strain for mouse exencephaly (SELH/Bc) that a high-fat diet increased the incidence of NTD (Harris and Juriloff, 2005). Kappen and Salbaum, working in a mouse model of diabetic embryopathy, also showed that intake of a high-fat diet tripled the incidence of NTD compared to a high-protein diet (Kappen et al., 2011). A recent study showed that feeding healthy rats an obesogenic high-fat diet increased the rate of developmental delay and embryonic malformations, including NTD (Alcala et al., 2021).

Our laboratory described that mouse embryos lacking Scavenger Receptor Class B type I (SR-B1 KO) exhibit a high penetrance of cranial NTD (Santander et al., 2013). Despite the neural malformation observed in SR-B1 KO embryos, SR-B1 protein is not present in the embryo during neural tube closure but is localized in one of the two layers of the yolk sac: the parietal yolk sac (pYS) (Santander et al., 2013; Hatzopoulos et al., 1998). This extraembryonic tissue comprises trophoblast giant cells (TGCs), the Reichert´s membrane, and parietal endoderm cells. TGCs derived from the trophectoderm of the blastocyst mediate attachment and invasion of the embryo during implantation and participate in the early exchange of nutrients and endocrine signals between the mother and the fetus before forming a mature placenta (Hu D. and Cross J. C., 2010). The roles of TGCs or the pYS during neurulation have been largely unexplored. By contrast, the visceral yolk sac (vYS), particularly its endoderm-derived cell layer, has proven to be essential for the absorption of nutrients by endocytosis during neurulation (Zohn and Sarkar, 2010). SR-B1 is a non-endocytic receptor involved in the bidirectional lipid exchange between high-density lipoproteins and cells (Rigotti et al., 2003). SR-B1 KO adult mice show vitamin E deficiency in several tissues, including brain, lungs and gonads (Mardones et al., 2002). We described that SR-B1 KO embryos also exhibit severe vitamin E deficiency and higher levels of reactive oxygen species (ROS) than their wild type (WT) littermates (Santander et al., 2017). Interestingly, feeding dams with a vitamin E-enriched diet completely rescued the NTD phenotype and reduced ROS levels in KO embryos, underscoring the relevance of this vitamin in neural tube closure (Santander et al., 2017). Vitamin E corresponds to α-tocopherol, which is the only member of the tocochromanol family that meets the requirements to be called a vitamin (Medicine, 2000). This fat-soluble micronutrient was first identified as a compound essential for rat fertility that protects membrane lipids from oxidative damage (Blaner et al., 2021) and also ehibits non-antioxidant molecular functions (Khadangi and Azzi, 2019).

Malnutrition by excess can alter the requirements for different micronutrients (Manna and Jain, 2015). Increased low-grade chronic inflammation, high oxidative stress and sequestration of certain micronutrients in adipose tissues have been proposed as potential mechanisms linking the chronic intake of hypercaloric diets and low micronutrient availability. In humans with obesity or metabolic syndrome, hyperlipidemia and lipoprotein oxidation can lead to the oxidation of α-tocopherol and reduce the bioavailability of this nutrient (Myara et al., 2003; Jessup et al., 1990; Gunanti et al., 2014). Other mechanisms, including reduced intestinal absorption (Mah et al., 2015), high hepatic retention (Violet et al., 2020) and impaired distribution due to excess circulating lipids (Traber et al., 2015) have also been suggested to contribute to lower circulating α-tocopherol levels. Whether a low vitamin E status increases the risk of NTD in women with obesity or malnutrition has not been addressed.

In this study, we studied if malnutrition by excess increased NTD incidence in SR-B1 KO embryos and evaluated the potential participation of lower maternal bioavailability of vitamin E as a mediator of this effect. Using pregnant females from HET intercrosses, we evaluated the effect of maternal intake of a high fat-high sugar (HFHS) or a vitamin E-supplemented HFHS diet (HFHS+VE) on NTD incidence, and studied different outcomes in dams, embryos and extraembryonic tissues that may account for those effects.

We found that preconceptional intake of the HFHS diet hampered the maternal vitamin E status and induced metabolic abnormalities in pregnant females, increasing the incidence of developmental delay and NTD, especially, but not exclusively, in SR-B1 KO embryos. Maternal vitamin E supplementation partially reduced HFHS-induced metabolic abnormalities and completely prevented embryonic malformations, possibly through indirect mechanisms involving lipid handling by the pYS.

## 2 Results

### 2.1 Vitamin E supplementation ameliorates HFHS-induced preconceptional metabolic dysregulation in females

**FIGURE 1:**
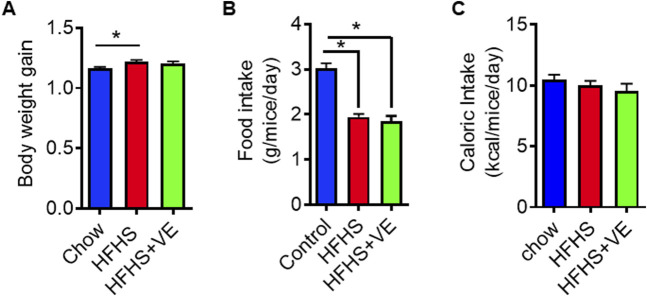
Weight gain and food intake in dams fed chow, HFHS and HFHS+VE diets. **(A)** preconceptional body weight gain: weight of females after/weight of females before administration of the diets for 8 weeks, **(B)** Food intake (g chow/mice/day) and **(C)** Caloric intake (kcal/mice/day). Different letters indicate statistical significance (p < 0.05 One-Way ANOVA, Tukey’s post-test, n = 10 animals per group).

SR-B1 HET females fed for 8 weeks before pregnancy a HFHS showed a small, yet significant, weight gain compared to HET females fed regular chow (Figure 1A). Females in the HFHS+VE group showed an intermediate weight gain. The discrete increase in weight observed in HFHS and HFHS+VE groups may be explained by the fact that females fed the hypercaloric diet consumed significantly less food than those in the chow group (Figure 1B). Indeed, the daily caloric intake was similar among the three groups (Figure 1C).

**FIGURE 2:**
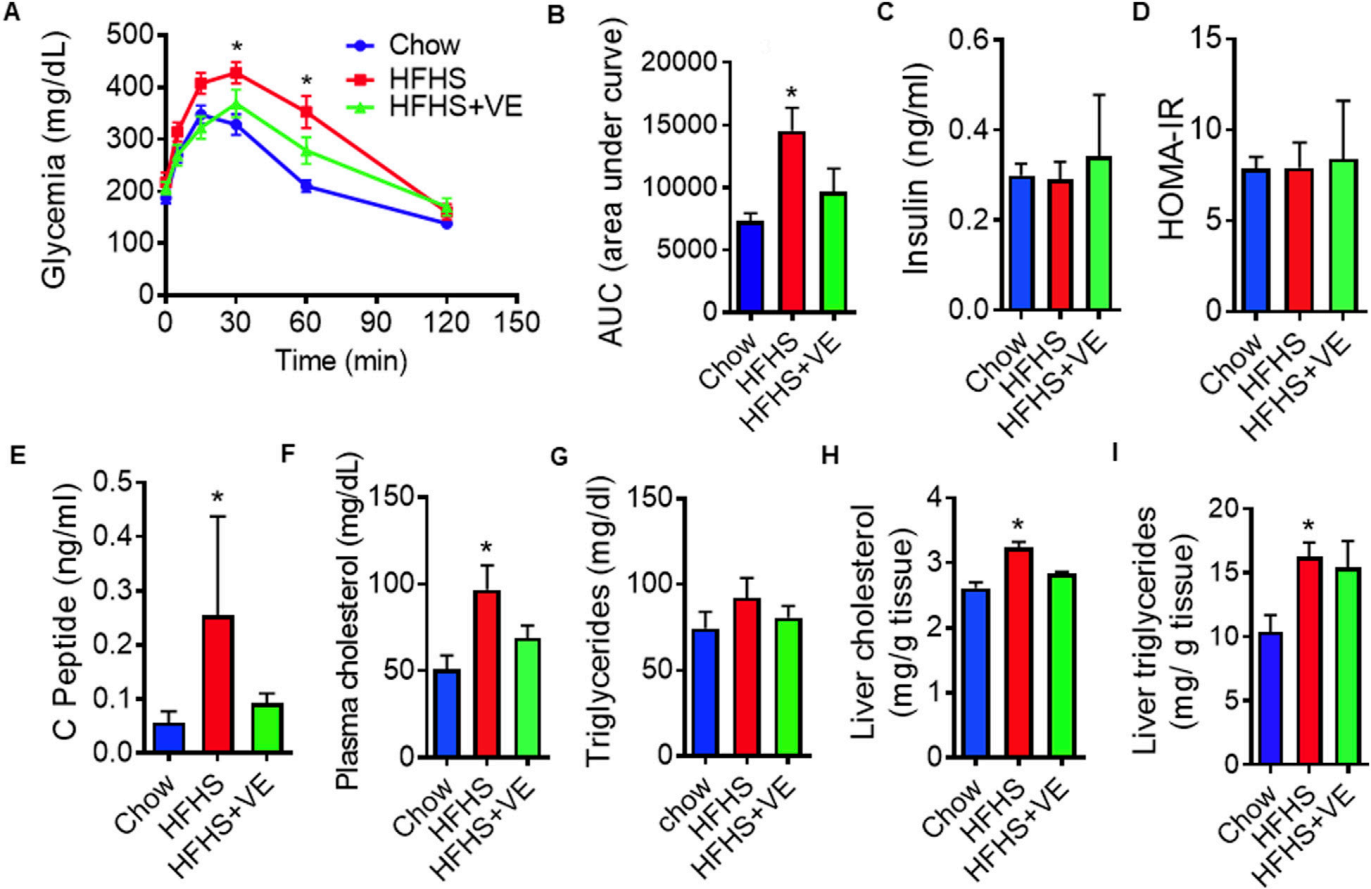
Metabolic dysregulation and effect of vitamin E on HFHS-fed females before mating. **(A)** Intraperitoneal glucose tolerance test (ipGTT) in dams fed chow, HFHS or HFHS+VE diets (n = 10 animals/group) and **(B)** Area under the glucose curve during i.p. GTT; **(C)** Fasting insulin plasma concentrations (n = 7), **(D)** Homeostasis Model Assessment (HOMA), used as an indicator of insulin resistance measured in mice at sacrifice (n = 7); **(E)** Fasting plasma concentrations of C-peptide (n = 7) and **(F)** Cholesterol (n = 7) **(G, H)** Hepatic cholesterol (n = 4) and **(I)** triglycerides levels (n = 7). Results are represented as mean ± SEM. Different letters indicate statistical significance (p < 0.05 One-Way ANOVA, Tukey’s post-test).

Compared to chow-fed females, females from the HFHS group showed glucose intolerance, as determined by higher plasma glucose levels at 30 and 60 min and higher area under the curve (AUC) (Figures 2A, B). Females in the HFHS+VE group showed an improvement in glucose tolerance, reaching intermediate glucose levels at 30 and 60 min and AUC in between that of the HFHS group and the chow group (Figures 2A, B). Unexpectedly, insulin levels (Figure 2C) and insulin resistance indexes (Figure 2D) were similar among the three groups. A higher circulating concentration of C-peptide, a byproduct of proinsulin cleavage used as a marker of endogenous insulin production, was observed in HFHS-fed dams (Figure 2E). Interestingly, vitamin E supplementation brought C-peptide levels to an intermediate state in HFHS-fed dams. Females fed the HFHS diet exhibited higher circulating cholesterol levels than dams fed chow (Figure 2F), accompanied by normal serum triglycerides (Figure 2G). HFHS dams also showed hepatic cholesterol and triglycerides accumulation (Figures 2H, I). In the HFHS+VE group, the circulating and hepatic lipid concentrations showed a partial reduction.

Altogether, these results showed that preconceptional feeding of SR-B1 HET females for 8 weeks with a HFHS diet induced metabolic alterations in mice, and that co-administration of vitamin E partially reduced those abnormalities.

### 2.2 Vitamin E supplementation prevents embryonic malformations caused by HFHS diet intake

**FIGURE 3:**
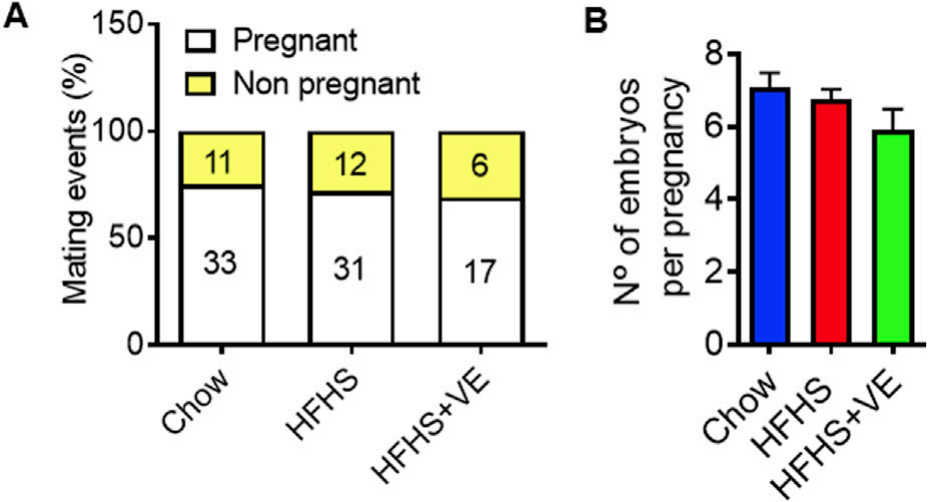
Effect of the diets on fertility. **(A)** Pregnancy rates as % effective pregnancy/plug detected (number of events inside graph bars). No differences were observed (Fisher´s test) and **(B)** Mean number of embryos per pregnancy. No differences were detected (ANOVA, followed by *post hoc* Tukey’s).

After the 2-month dietary intervention, females were caged with HET males. Intake of the HFHS diet alone or supplemented with vitamin E did not affect the fertility of the females, as shown by similar pregnancy rates (Figure 3A) and mean number of embryos (Figure 3B) than those in control, chow-fed dams.

**FIGURE 4:**
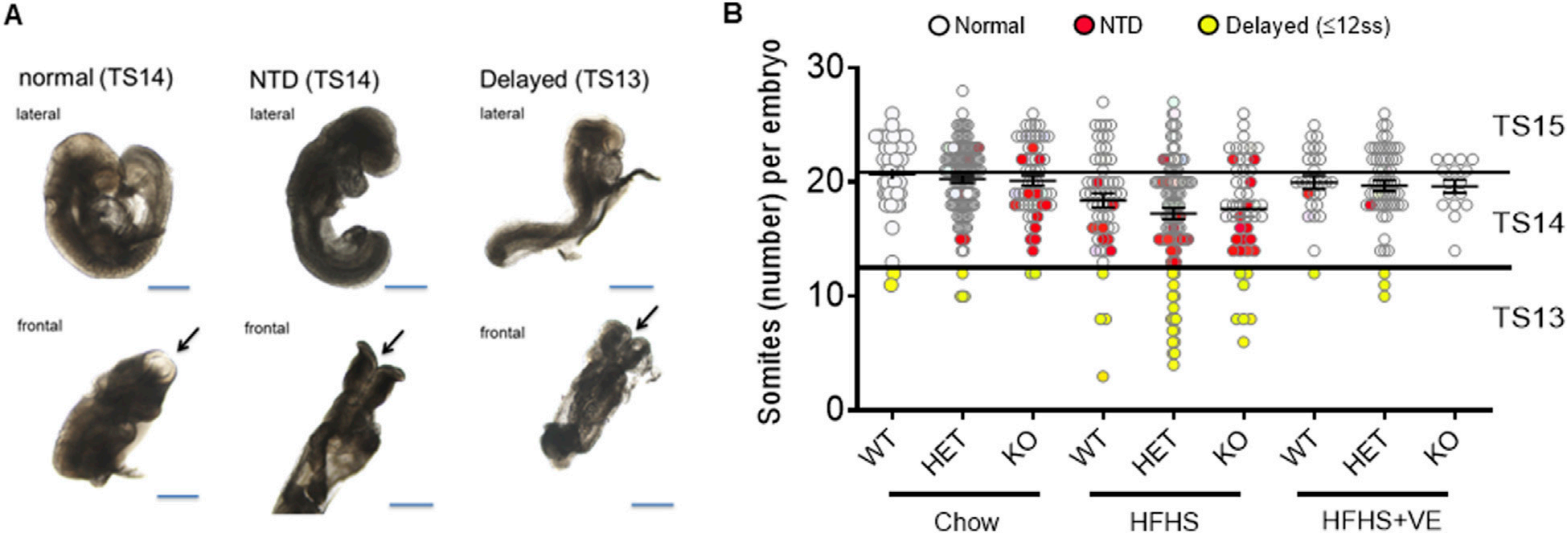
Different phenotypes in embryos retrieved from pregnant dams. **(A)** Representative images of embryos, in lateral and frontal views, showing a normal morphology, with complete neural tube closure and expected number of somites (left panel), an open neural tube (NTD) and normal number of somites (middle panel) or open neural tube, low number of somites (delayed) and no embryo-turning along the axis (right panel). Arrows indicate the anterior neuropore. Bar: 500 μm. **(B)** Individual number of somites/embryo according to maternal diet and genotype. The straight lines separate the Theiler Stages: TS15 (21–29 somites), TS14 (13–20 somites), TS13 (less than 13 somites). Embryos with a closed neural tube are represented as white dots, embryos with NTD as red dots, and developmentally delayed embryos as yellow dots.

At E9.5, embryos were retrieved from dams in the different groups and, after genotipification, they were classified according to their genotype, morphology and somite number. All the embryos were alive, evidenced by a beating heart, when they were dissected. They also showed normal development of the branchial arches and the heart, as well as initial differentiation of the optic vesicles and early signs of limb development (Figure 4A). As expected, at that gestational stage most embryos had more than 13 somites, which corresponds to Theiler stages 14–15 (TS14 and TS15) (Davidson and Theiler, 1989) (Figure 4B). A proportion of embryos were underdeveloped for E9.5, especially in pregnancies from the HFHS group. All these embryos had an open neural tube but, as they did not reach the developmental stage expected for E9.5 (had less than 13 somites), they were classified in a different category which we denominated “developmentally delayed”.

**FIGURE 5:**
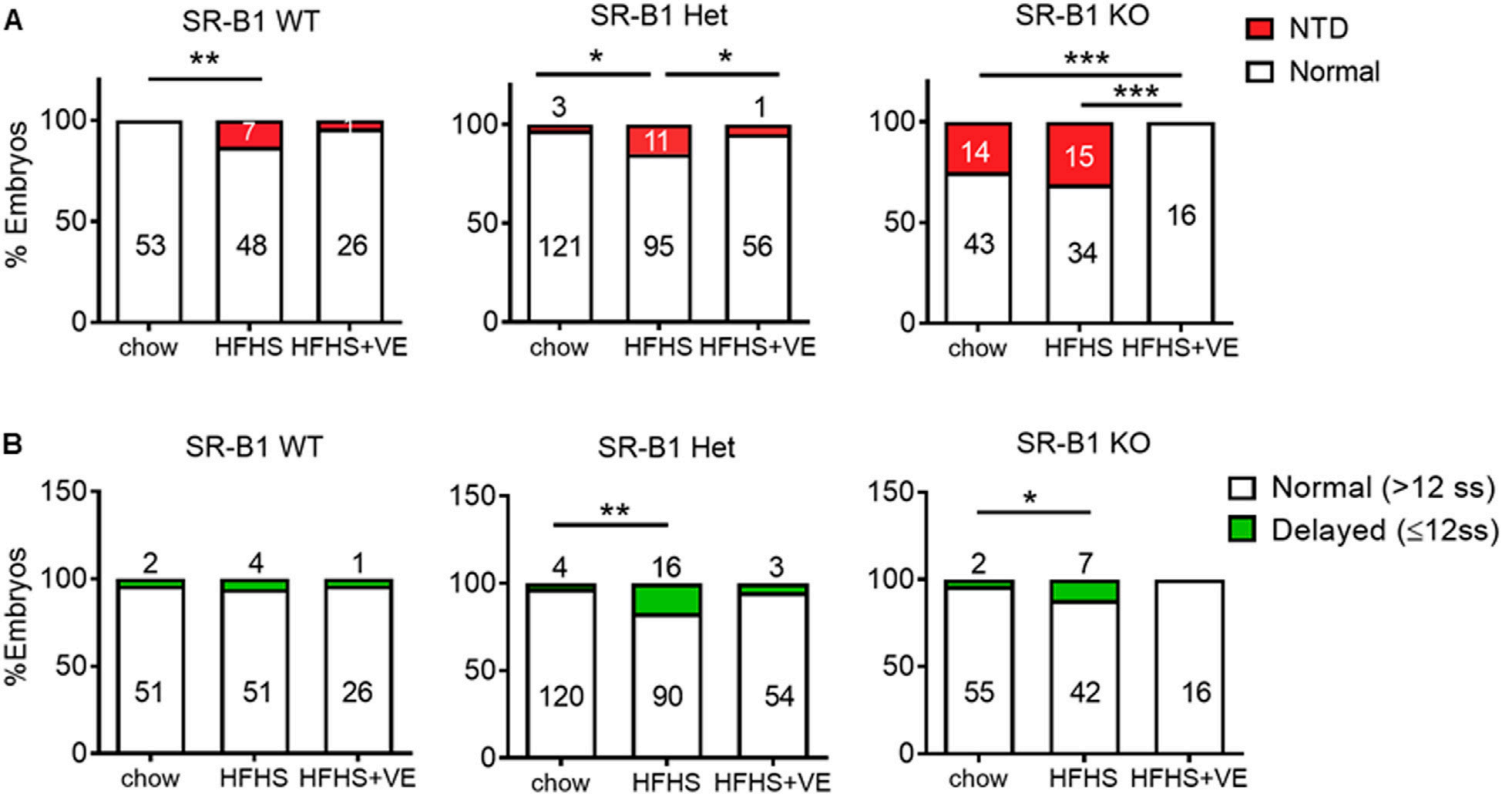
Effect of HFHS intake and vitamin E supplementation on malformation rates in embryos. **(A)** Graphs showing proportions of morphologically normal (white) and NTD (red) embryos for each SR-B1 genotype and maternal diet. The number of embryos in each category is shown inside the bars. **(B)** Proportions of normal (white) vs. developmentally delayed (green) embryos for each SR-B1 genotype and maternal diet. Statistical analyses were performed by comparing the proportions of embryos (*p < 0.05, **p < 0.01, ***p < 0.001, χ^2^ test).

SR-B1 KO embryos with appropriate development for E9.5 from chow-fed dams exhibited an NTD ratio of 25% (Figure 5A). In agreement with our previous studies (Santander et al., 2013), this malformation was infrequent (less than 2%) in WT and HET embryos from females fed the control diet. In SR-B1 KO embryos from the HFHS group, the rate of NTD increased to 31%. Unexpectedly, around 12% of SR-B1 WT and HET embryos in HFHS-fed dams exhibited NTD. Maternal vitamin E supplementation of dams fed the HFHS diet reduced NTD incidence in embryos from all the genotypes. Although this reduction was only significant in HET and KO embryos, only 4% of WT, 2% of HET, and 0% of KO embryos were malformed in dams fed the HFHS+VE diet. As described previously, NTD in SR-B1 KO embryos was sex-dimorphic, with a higher incidence in female embryos from both chow-fed and HFHS-fed dams (Supplementary Figure 1).

The preconceptional intake of the HFHS diet significantly increased the number of developmentally delayed embryos, i.e., smaller than 13 somites, which had still not undergone embryonic turning nor neural tube closure, from the HET and KO genotypes (Figure 5B). Notably, vitamin E supplementation reduced the rates of developmental delay to levels found in chow-fed dams. The occurrence of developmental delay was not concentrated in certain specific litters, as shown in 10 representative pregnancies (Supplementary Figure 2).

In sum, neither a HFHS diet nor vitamin E supplementation affected female fertility, but the HFHS diet increased the incidence of developmental delay and NTD in embryos, especially from the SR-B1 KO and HET genotypes. Maternal vitamin E supplementation reduced NTD incidence and developmental delays across all genotypes to levels similar to those in chow-fed dams, suggesting its protective role against the negative effects of the HFHS diet.

### 2.3 Reduced vitamin E bioavailability and abnormal cholesterol handling in the parietal yolk sac may contribute to modulating the risk of HFHS-induced embryo malformations

**FIGURE 6:**
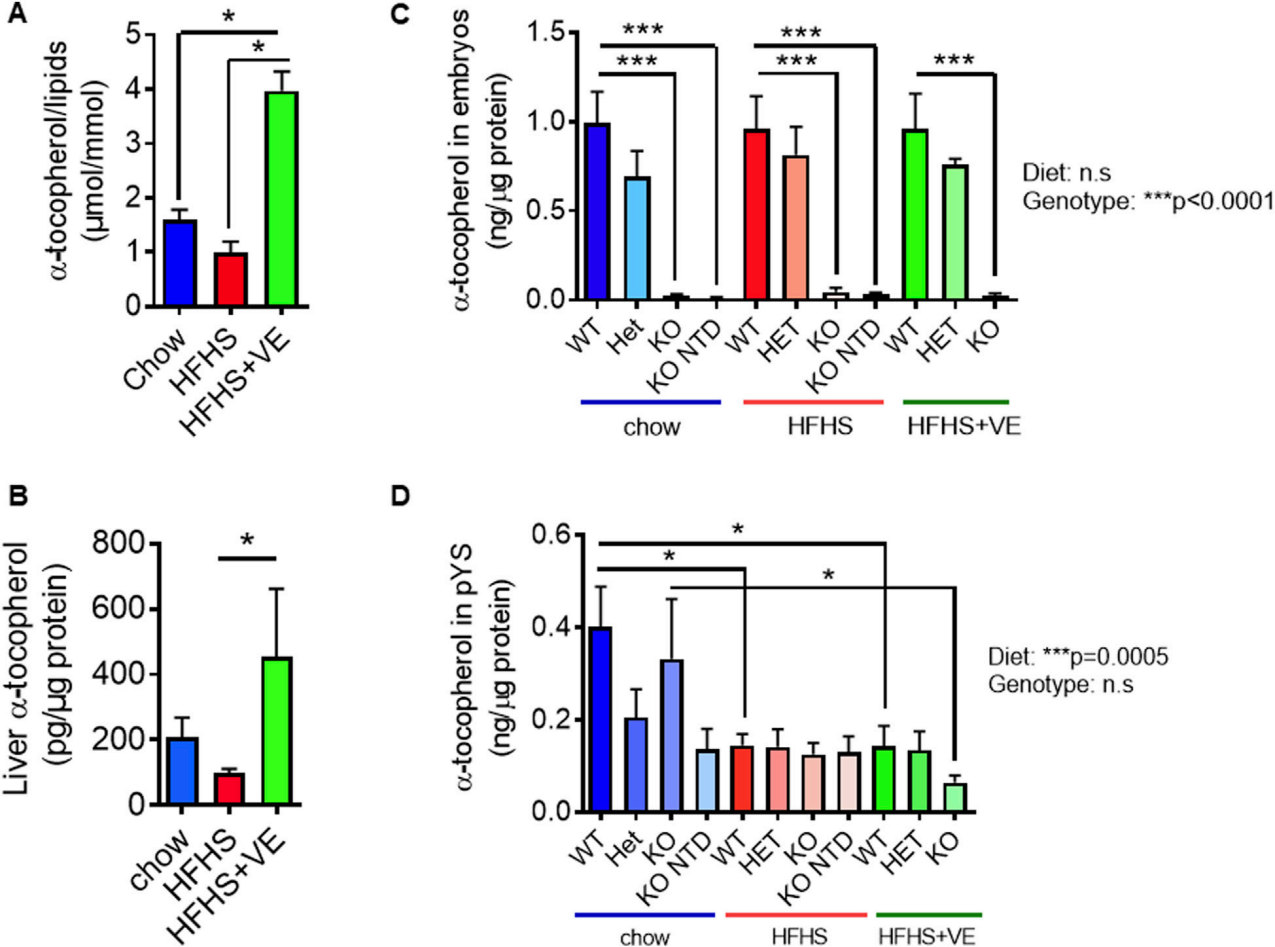
Impact of the HFHS diet on maternal, embryonic and extraembryonic vitamin E levels at E9.5. Maternal vitamin E levels were determined in **(A)** plasma (corrected by the addition of cholesterol and triglycerides levels) and **(B)** liver of pregnant animals from all experimental groups (One-Way ANOVA, Dunn’s post-tests, n = 5 animals per group). Vitamin E levels in **(C)** parietal yolk sacs (n = 5-9 samples per group) and **(D)** embryos (n = 3). Statistical analyses were performed by Two-way ANOVA (p values for diet and genotype effects are shown next to graphs) and Bonferroni’s post-test (*p < 0.05 and ***p < 0.001).

We next assessed the vitamin E status of pregnant females from the different groups. We determined the levels of α-tocopherol in plasma and liver homogenates at E9.5. Plasma α-tocopherol levels were normalized to the levels of circulating lipids, as this correction allows a more appropriate assessment of vitamin E status in patients with obesity-associated hyperlipidemia (Traber, 2014). Pregnant females in the HFHS group exhibited a ∼40–50% reduction in serum α-tocopherol/lipid and hepatic α-tocopherol levels compared to dams in the chow group (Figures 6A, B), although this reduction was not statistically significant and was not associated to an increase in systemic or hepatic oxidative stress in the females (Supplementary Figure 3). As anticipated, vitamin E supplementation resulted in significant increases in circulating and hepatic vitamin E contents.

We previously reported that SR-B1 KO embryos show severe vitamin E deficiency (Santander et al., 2017). In this study, we observed that vitamin E levels in SR-B1 KO embryos from the chow, HFHS and HFHF + VE dams were barely detectable (Figure 6C). Maternal supplementation with vitamin E of dams fed the HFHS diet did not reestablish the levels of vitamin E in SR-B1 KO embryos (Figure 6C), as previously reported in chow-fed dams (Santander et al., 2017). Considering that giant trophoblast cells from the pYS express high levels of SR-B1 (Santander et al., 2013) and, taking into account that this extraembryonic tissue is involved in the regulation of immune tolerance and the materno-embryonic provision of nutrients during embyo development (Hu D. and Cross J. C., 2010; Burton et al., 2001; Cindrova-Davies et al., 2017), we evaluated the vitamin E content in this extraembryonic tissue (Figure 6D). Regardless of the SR-B1 genotype, pYS from dams fed the HFHS diet showed lower vitamin E levels, and those levels were not restored upon maternal supplementation with vitamin E (Figure 6D).

**FIGURE 7:**
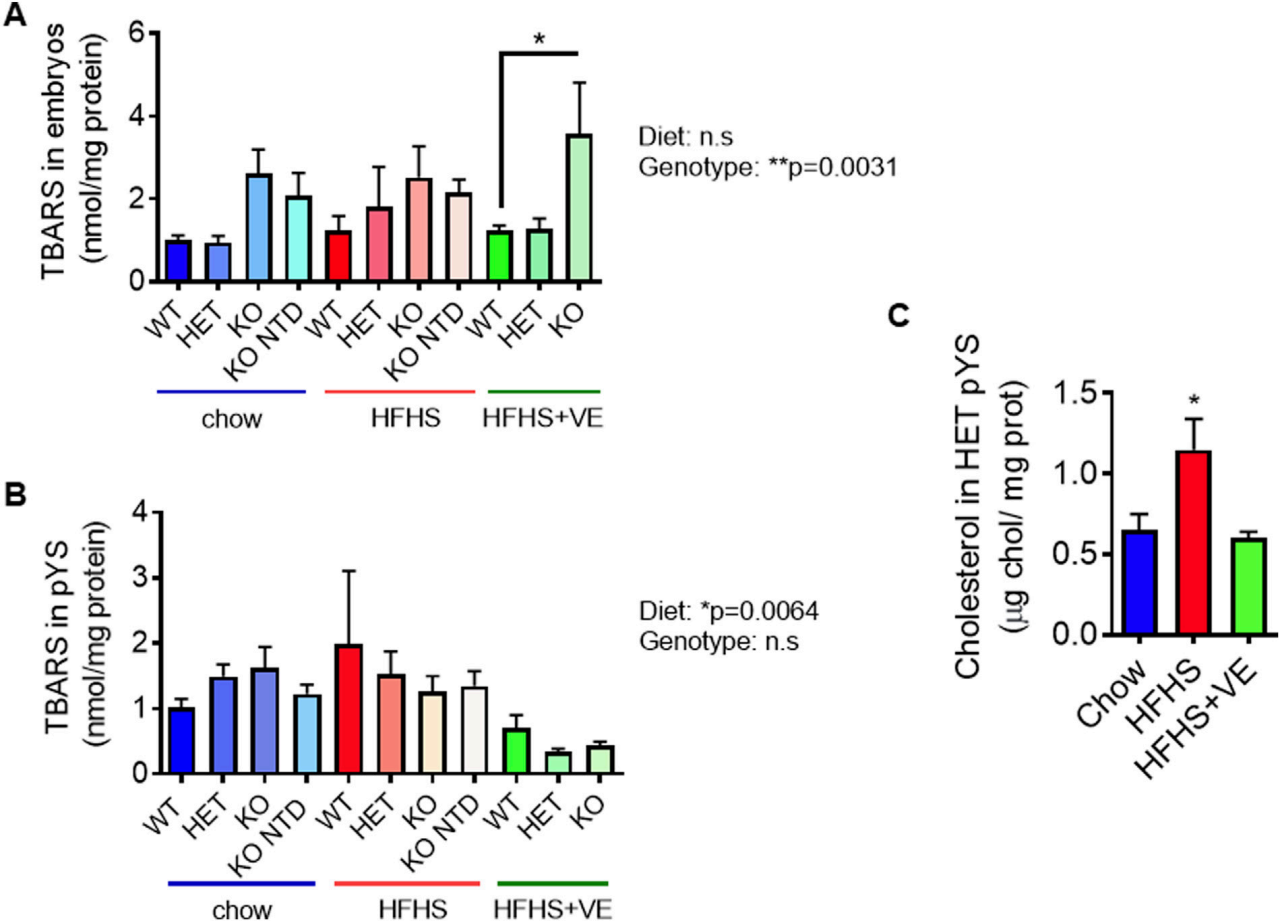
Impact of the HFHS diet on embryonic and extraembryonic lipoperoxidation at E9.5. **(A)** Determination of lipoperoxidative damage through TBARS levels in pools of 2 embryos (n = 6–9/group) and **(B)** individual pYS samples per group (n = 3–5/group) for each SR-B1 genotype and maternal diet group. Two-way ANOVA (P values for diet and genotype effects are shown next to graphs) and Bonferroni’s post-test (*p < 0.05). **(C)** Cholesterol levels in heterozygous pYS from dams fed the different diets (*p < 0.05).

Considering that SR-B1 KO embryos are severely vitamin E deficient, that pYS from HFHS-fed dams exhibit lower vitamin E levels, and taking into account the antioxidant role attributed to vitamin E, we next sought to assess the impact of lipid oxidative damage in both embryonic and extraembryonic tissues from the different SR-B1 genotypes and maternal diets, by measuring thiobarbituric acid reactive substances (TBARS) concentrations. Overall, embryonic lipid peroxidation was not influenced by the diets but was affected by SR-B1 deficiency, although pairwise comparisons did not show significant differences with the exception of WT vs. KO embryos from the HFHS-fed group (Figure 7A). By contrast, in pYS, a significant effect of TBARS levels caused by the diet, and not by the genotype, was observed (Figure 7B). Although no significant differences were observed between genotypes of different diets, the pYS of all the genotypes from the HFHS + VE showed a global TBARS reduction.

As shown previously, plasma and livers from dams fed the HFHS diet show high cholesterol levels which are partially prevented by vitamin E supplementation. To evaluate if cholesterol in pYS mirrored the rise in maternal plasma and liver cholesterol due to HFHS intake, we used heterozygous pYS from the three experimental groups. Results showed that maternal intake of the HFHS diet led to a significant increase in cholesterol content in pYS (Figure 7C), which was prevented by co-administration of vitamin E. This result suggests a systemic protective effect of vitamin E on cholesterol metabolism in the parietal yolk sac.

In sum, our results show that consumption of a HFHS tended to reduce serum and hepatic vitamin E levels. As shown previously in chow-fed pregnant females, SR-B1 KO embryos exhibited severe vitamin E deficiency regardless of the maternal diet or supplementation, and maternal vitamin E supplementation did not restore vitamin E levels in these embryos. Lipid peroxidation levels, as measured by TBARS, were influenced by diet in extraembryonic tissues but not in embryos, with HFHS + VE group showing a reduction in TBARS levels compared to the HFHS group without supplementation. Altogether, these results suggest that HFHS may alter maternal vitamin E biovailability and that the prevention of NTD by vitamin E supplementation is mediated by indirect mechanisms, as vitamin E levels in the embryo itself do not explain the effect. Our preliminary results show that one potential mechanism through which diet may modulate NTD is the modulation of oxidative stress and cholesterol content in the pYS.

## 3 Discussion

The administration of the HFHS to female mice induced metabolic abnormalities, as previously observed in numerous studies (Doulberis et al., 2020). The metabolic phenotype resulting from the dietary intervention with the obesogenic diet was moderate. The females did not show fasting glycemia or insulinemia, and their fertility was completely normal. However, they were glucose intolerant and showed and higher plasmatic levels of C-peptide, which suggest the occurrence of previous or transient hyperinsulinemia, suggestive of reduced insulin sensitivity. The dams also exhibited high circulating and hepatic lipid levels, which usually occur concomitant to insulin resistance due to increased lipolysis and *de novo* lipogenesis.

We hypothesized that consumption of a HFHS diet would alter the vitamin E status in pregnant dams. Plasma α-tocopherol levels in mice fed the HFHS diet were analyzed in relation to cholesterol and triglyceride concentrations because it has been demonstrated that vitamin E levels and antioxidant capacity is determined by the levels of circulating lipids and the amount of adipose tissue (Violet et al., 2020; Traber, 2014). Although we did not find statistically significant differences, we observed a tendency towards lower levels of plasma α-tocopherol in relation to lipids in females of the HFHS group. Regarding hepatic vitamin E measurements, we normalized the values by tissue mass because there is no consensus on whether to correct this parameter by tissue lipid levels and also found a tendency to lower levels in HFHS-fed dams (Traber, 2014). However, as the livers of mice consuming the HFHS diet show lipid accumulation, the hepatic requirement for vitamin E to protect lipids from oxidation is likely to be higher compared to the control group. Our results showed, however, that the mice fed the HFHS diet did not show an altered oxidative status before pregnancy.

Our results showed that co-administration of vitamin E with the HFHS diet had a beneficial effect on all the altered metabolic parameters in pregnant dams. Alpha-tocopherol has previousy been shown to improve insulin resistance and dyslipidemia in human overweight patients, through reduction of oxidative stress, improvement of the hepatocellular function, and a transient effect on insulin resistance (Manning et al., 2004). In male mice, the oral administration of α-tocopherol with a high-fat diet had positive effects on insulin resistance, lipid profiles, and oxidative stress through the expression of PPAR-α and PPAR-γ (Kim et al., 2013). Vitamin E can also modify the expression of genes encoding key enzymes related to endogenous cholesterol biosynthesis by decreasing the transcription of the SREBP-2 factor (Landrier et al., 2010; Valastyan et al., 2008). Vitamin E supplementation is an effective treatment for non-alcoholic fatty liver disease (NAFLD) through reduction of liver oxidative damage, steatosis and inflammation (Hoofnagle et al., 2013; Sanyal et al., 2010; Podszun et al., 2020).

In our study, around 15% of HET and KO embryos from dams fed the HFHS diet showed developmental delay, a phenotype that was only observed sporadically in embryos from the chow-fed group or in WT embryos from HFHS-fed dams. Developmental delay has been reported as an adverse consequence of an altered maternal metabolism in mouse models of diabetes. Embryos from diabetic mothers have been proposed to respond to hyperglycemia either by suppressing gene expression in a generalized manner and undergoing developmental delay, or by overexpressing compensatory genes that allow them to grow normally, but develop NTD - or other malformations – at later stages (Zhao et al., 2016; Zhao et al., 2017). Exposure to hyperglycemia during early organogenesis in mice has also been shown to alter gene expression in a stage-dependent manner: on day E8.5, the genes affected by hyperglycemia are mainly involved in cell proliferation, whereas from day E9.5 onwards, the affected genes are more related to cytoskeletal organization, oxidative phosphorylation, cell migration, and cell differentiation. Although the relationship between high cholesterol or triglyceride levels and the increased incidence of NTD has been proposed (Zhao et al., 2016; Zhao et al., 2017), the participation of maternal hyperlipidemia as an independent risk factor for NTD has not been demonstrated up to date. Embryonic developmental abnormalities have been described in mouse mutants deficient in different proteins involved in lipoprotein metabolism, most of which are expressed in the vYS, i.e., cubilin (Perea-Gomez et al., 2019), LRP2/Megalin (Spoelgen et al., 2005), ApoB (Homanics et al., 1995) and MTTP (Raabe et al., 1998), suggests that the maternal provison of lipids may be relevant during organogenesis. In this context, a key strength of our study is that it demonstrates the essential role of a lipid receptor expressed in the parietal yolk sac (pYS) for successful neurulation. Given that stages E7.5 and E8.5 are the most sensitive to maternal metabolic abnormalities, transient hyperglycemia and/or hyperlipidemia during this time window may have caused developmental delay and increased NTD risk in a proportion of embryos from HFHS-fed dams. Indeed, the administration of the HFHS diet also caused an increase in NTD in embryos with adequate development from all the genotypes.

Our results showed that the maternal intake of the vitamin-enriched diet completely prevented developmental delay and NTD in embryos of all the genotypes from HFHS dams. We showed previously that the preventive effect of vitamin E on NTD in SR-B1 KO embryos was not mediated by a normalization of the levels of vitamin E in embryos (Santander et al., 2017). This also seems to be the case in NTD due to maternal HFHS intake, as the levels of α-tocopherol were similar between embryos from the HFHS and HFHS-VE groups. Accordingly, maternal vitamin E supplementation did not reduce TBARS levels, which are increased in SR-B1 KO embryos, independently of the maternal diet. The HFHS diet did not directly impact embryonic vitamin E levels but reduced vitamin E content in the pYS. Although vitamin E levels were not increased in the pYS in embryos from the HFHS-VE group, this intervention reduced lipoperoxidation and prevented cholesterol accumulation in this extraembryonic tissue. These results suggest that the pYS may be influenced by the maternal environment and that dysregulation in its metabolism could impact neural tube closure in embryos. In this scenario, vitamin E may contribute to the prevention of NTD by protecting the pYS. This extraembryonic tissue contributes to the exchange of nutrients and gases between the mother and fetus in the early post-implantation conceptus (Hu D. and Cross J., 2010). We previously showed that TGCs from the pYS express several proteins involved in lipoprotein metabolism, such as the HDL receptor Gpihbp1, receptors for other lipoproteins (e.g., Ldlr, Apobr, and Lrp1), and the lipid transporters Abca1 and Abcg1, which mediate lipid efflux from cells to Apoa1 and HDL, respectively, in addition to SR-B1, suggesting that they may active in taking up and potentially transferring lipids from the mother to the conceptus (Santander et al., 2017; Hannibal et al., 2014). The role of extraembryonic tissues in determining embryonic developmental fate was described decades ago and has gained relevance in the last few years, particularly in the visceral yolk sac in mouse models of diabetic embryopathy. Visceral yolk sacs from rat conceptuses cultured under hyperglycemic conditions show an altered ultrastructure, with blunt microvilli, increased apical liposomes, less endoplasmic reticulum and lower number and size of lipid droplets (Pinter et al., 1986a), associated with higher free fatty acids and triglycerides (Pinter et al., 1988). Interestingly, NTD and abnormal lipid content in the yolk sac due to hyperglycemia can be prevented by the inclusion of arachidonic acid in the culture medium, suggesting that aberrant accumulation of neutral lipids in these membranes may be a mediator of embryonic malformations (Pinter et al., 1986b). In a recent study, C Kappen and her group used two independent mouse models of maternal diabetes and a high-fat diet-feeding model of maternal obesity, and observed excessive lipid accumulation at 8.5 days in the visceral yolk sac (Zhang et al., 2023). In the three models, a low association of lipid droplets with lysosomes was observed, what led the authors to propose that defective lipid processing in extraembryonic tissues from pregnancies affected by maternal metabolic disease may lead to reduced availability of lipids to the developing embryo. The former evidence, together with the results obtained in our study, highlight the role of extraembryonic tissues during neural tube closure and propose that the preventive effect of vitamin E on NTD may be mediated by protecting the pYS from oxidative damage and aberrant lipid accumulation.

A limitation of this study is that we used a non-purified chow diet as a control, whereas the HFD was purified. However, both diets have similar protein, vitamin, and mineral content and the main difference was in the calories/kg and the macronutrients providing that energy: carbohydrates in the former and fats in the latter. In addition, both diets were formulated to ensure adequate vitamin intake in mice. Also, although they differ slightly in their vitamin E content, this difference does not seem to explain the deficient loading of vitamin E in pYS because, otherwise, this would have been prevented by feeding dams the HFHS-vitamin E supplemented diet, which has significantly higher vitamin E levels. However, an insufficient provision of other micronutrients relevant for neurulation cannot be overruled in embryos from females fed the HFHS diet, because they consumed significantly less food than those in the chow group.

The fact that vitamin E levels in SR-B1 KO embryos were not restored by maternal supplementation of dams with this vitamin, despite the significant preventive effect of this intervention, is intriguing and a current matter of research in our group. It may be proposed that high oxidative stress in SR-B1 KO embryos due to their very low vitamin E content may also affect the availability of other nutrients or metabolities relevant for neurulation previously shown to be affected by reactive oxygen, i.e., betaine and other methyl donors (Kreuz and Fischle, 2016; Zhang et al., 2020; Head and Traber, 2021).

Although intake of vitamin E-deficient diets have been associated with miscarriage (Shamim et al., 2015; Simşek et al., 1998) and intrauterine growth restriction (Kositamongkol et al., 2011), the α-tocopherol status is not frequently evaluated in women of reproductive age or during pregnancy. Treating pregnant women with high doses of vitamin E has proven to be relatively safe throughout the second and third trimesters, although not effective at preventing stillbirth, neonatal death, preterm birth, pre-eclampsia, term prelabour rupture of membranes or poor fetal growth (Rumbold et al., 2015). To date, there are no reports of ongoing or concluded clinical trials to assess the effect of maternal vitamin E supplementation on NTD prevention in humans. Despite geographical distances and cultural differences, vitamin E inadequacies of similar magnitudes have been detected in women of reproductive age from the United States (Maras et al., 2004), Spain (Olza et al., 2017) and Latin America (Busso et al., 2021). Thus, promoting the consumption of foods rich in vitamin E - such as vegetable oils, nuts, whole grains, and leafy green vegetables - in women of childbearing age may serve as a strategy to prevent NTD, especially in patients with metabolically compromised maternal conditions such as obesity or diabetes.

### 3.1 Concluding remarks

In a murine NTD model due to SR-B1 deficiency, administration of a HFHS diet altered glucose metabolism and induced a hyperlipidemic state in the mother, associated with an increase in the incidence of developmental delay and NTD in the embryos. Parietal YS from HFHS-fed dams showed lower vitamin E levels and cholesterol accumulation. Maternal supplementation with vitamin E completely prevented the embryonic abnormalities and partially or completely corrected the maternal and extraembryonic parameters induced by intake of the HFHS diet. Further studies will be aimed at understanding if the preventive effect of vitamin E on NTD is mediated by protecting the pYS – and potentially the vYS - from aberrant lipid handling due to oxidative damage.

## 4 Materials and methods

### 4.1 Animals and diet

We used mice in a mixed C57Bl6/J:129 background carrying a targeted mutation in the SR-B1 locus, donated to D. Busso by A. Rigotti and M. Krieger^37^. Animals were maintained in the animal facility of the Biomedical Reseach and Innovation Center (School of Medicine, Universidad de los Andes) at 25°C and 12 h light:dark cycling fed *ad libitum*. Protocols were conducted in agreement with the National Research Council (NRC) publication Guide for Care and Use of Laboratory Animals (copyright 1996, National Academy of Science). Studies were approved by IACUC from Universidad de los Andes (CEC2022030).

Two-month-old SR-B1 HET females were randomized into 3 groups and received the diets and water *ad libitum* for 8 weeks before mating. The control group (chow, n = 33) received a standard chow diet that provides 15% calories from fat (Prolab RMH3000, Labdiet; 75 IU vitamin E/kg) and normal tap water. The HFHS group (n = 31) received a diet that provides 60% calories from fat [58Y1 Rodent Purified Diet (TestDiet)] and sucrose water (45% fructose/55% glucose, 42 g/L). The vitamin E-supplemented group (n = 17) was fed the same HFHS diet but supplemented with α-tocopherol (as DL-alpha-tocopheryl acetate) [2.000 I.U. of α-tocopherol/kg diet; LT541 Rodent Purified Diet (TestDiet)]. This dose of vitamin E supplementation was previously shown to prevent NTD in SR-B1 KO embryos from chow-fed dams (Santander et al., 2017). Detailed information on the diets is described in Supplementary Table 1. Food intake and the weight of the animals were recorded weekly. The diet was kept at −20°C until use and feed in the cages was changed every 2 to 3 days. After mating, mice from all groups were fed the control chow diet, but vitamin E content was maintained in the supplemented group.

### 4.2 Retrieval of (E0.5) embryos and morphological analysis

To generate intercrosses, 2- to 4-month-old HET females were caged with 2-to 6-month-old SR-B1 HET males at a 1:1 or 2:1 ratio. Female mice were checked daily for the presence of a vaginal plug during the first hour of the light cycle. The day a plug was detected was recorded as embryonic day 0.5 (E0.5) and the female was separated from the male and maintained with one or two females in the same cage. All embryos were collected on day E9.5 when neural tube closure is complete in wild-type embryos. Pregnant dams were anaesthetized with a mixture of ketamine:xylazine (0.18 mg:0.012 mg per gram of body weight) and the peritoneal cavity was exposed. A blood sample was taken from the abdominal vena cava and the uteri were excised. Implantation sites were retrieved and the embryos and pYS were collected. Embryos were assessed for neural tube closure and classified into normal or NTD and somite number was determined. The visceral yolk sac was snap-frozen in liquid nitrogen, preserved at −80°C and used for individual genotyping, as described previously (Santander et al., 2013). Embryo and PYS samples were also immediately frozen in liquid nitrogen and stored individually at −80°C until use. As each dam produced variable numbers of embryos with different genotypes and phenotypes, pools of embryos retrieved from different dams were used in some experiments. For all analyses of vitamin E and oxidative damage markers, we selected embryos of similar size and collection time to avoid differences resulting from analyte instability.

### 4.3 Metabolic parameters

#### 4.3.1 Glucose metabolism

At the end of dietary treatments, mice were fasted for 6 h. For glucose tolerance test, after measuring the baseline blood glucose concentration from a tail cut by a Glucometer test strip (Freestyle Optium, United Kingdom) mice were injected intraperitoneally with 100 mg/mL glucose at 1 mg/g body weight. Blood glucose concentrations were then measured at 15, 30, 60, and 120 min after glucose injection. Insulin and C-Peptide were assayed by enzymatic colorimetric tests (EZRMI-13K, Merck, Germany; MBS2567399, Mybiosource, United States). The C- Peptide is a part of insulin that is used as a marker of previous chronic or transient hyperinsulinemia because it remains in circulation longer than insulin due to renal instead of hepatic catabolism. The HOMA-IR (homeostasis model assessment of insulin resistance) index was calculated as [fasting serum glucose × fasting serum insulin/22.5] to assess insulin resistance.

#### 4.3.2 Lipids

At the end of dietary treatments, mice were fasted for 6 h. Plasma was separated by centrifugation at 4,000 rpm for 20 min at 4°C and lipid extraction was performed using methanol:chloroform 1:2 following standard procedures. Cholesterol was measured by an enzymatic assay (Allain et al., 1974) and tryglicerides were measured using the VITROS TRIG Slides XT3400 technology (Ortho Clinical Diagnostics, United Kingdom) by an external service (Barnafi-Krause Laboratory, Chile). In pYS, the cholesterol content was determined using the Amplex Red Cholesterol Assay (Invitrogen), because of a higher sensitivity of this test, following the manufacturer’s instructions. Briefly, extracts were dissolved in reaction buffer and incubated with cholesterol oxidase, peroxidase and the Amplex Red reagent. The enzymatic oxidation of cholesterol drives the stoichiometric oxidation of the Amplex Red reagent to resorufin. The fluorescence of the product was measured on a microplate fluorimeter with excitation at 530 nm and emission reading at 590 nm. Results were expressed as nanogram of cholesterol per microgram of protein in the lysate. In each experiment, a known quantity of pure cholesterol was processed in parallel to the samples for correction because of extraction efficiency.

### 4.4 Vitamin E

α-Tocopherol detection by HPLC was performed by an external service (Barnafi Krause Laboratory, Chile) using chromatographic methods as routinely employed. Briefly, pairs of embryos or single pYS or liver tissue (100 mg) were lysed in buffer (T-PER, Invitrogen) containing 1%N-acetylcysteine and centrifuged at 12,000 g at 4°C for 10 min. Lysates, as well as whole maternal plasma (200 μL) were mixed with a commercial mix of solvents (Chromsystems, Germany) and centrifuged at 9,000 g for 10 min. The supernatants were subjected to HPLC using an isocratic column (Chromsystems, Germany) and elution signal was detected with a UV detector (Shimadzu, Japan) initially at λ = 325 nm and then for 3.5 min at λ = 295 nm. The samples were run in duplicate and the signal was interpolated in a standard curve for α-tocopherol.

### 4.5 Lipid peroxidation

Thiobarbituric acid reactive substances (TBARS) is a well recognized parameter for lipid peroxidation levels which is also as a biomarker of tissue damage caused by oxidative stress. Briefly, pairs of embryos, single pYS, 10 µL of plasma or 100 mg of liver homogenate were lysed in 50 µL of RIPA buffer and mixed with thiobarbituric acid (TBA) at a ratio of 1:3.75:1.25 (sample:TBA:H2O). TBA was previously dissolved in water (0.8%) that had already been mixed with 20% acetic acid (pH 3,5) at a ratio of 1:1 and the mixture was then heated at 95°C for 2 h. After cooling, a mixture of n-butanol and pyridine (15:1 v/v) was added to each simple at a ratio of 1:1.25. The mixture was gently vortexed, centrifuged at 1.000 *g* for 10 min and the fluorescence of the upper phase was measured (530/550 nm) in a Tecan infinite M1000 PRO reader (Thermofisher, United States). Each reaction was performed in duplicate. The amount of TBARS was determined by the malondialdehyde standard curve and expressed as nmol/mg protein.

### 4.6 Sex determination

Individual sexing of embryos was performed by allele discrimination using PCR, as described previously^42^. The primer sequences F: 5′-CCG​CTG​CCA​AAT​TCT​TTG​G-3 and R: 5′-TGAAGCTTTTGGCTTTGAG-3′were used to amplify the bands corresponding to the smcx and y alleles in the X and Y chromosomes, respectively.

### 4.7 Statistic analyses

Results are presented as mean ± SEM. For each variable, the normality of the distributions was assessed using the Kolmogorov–Smirnoff test. For Gaussian distributions, one-way analysis of variance (ANOVA), followed by *post hoc* Tukey’s or Bonferroni multiple comparison tests, were applied. Otherwise, the Kruskall-Wallis and Dunn’s multiple comparison test were used. When multiple variables were present, two-way analysis of variance (2-ANOVA) was used followed by *post hoc* Bonferroni’s test. The statistical significance of the difference between proportions was evaluated with the Fisher’s exact test. For maternal diet, SR-B1 genotype, and embryo morphology correlation, an additive score was used to convert each categorical variable into a discreet one. Then the Spearman’s Rank Correlation Coefficient was used to assess the relationship between NTD incidence and Rank (the sum of maternal diet and SR-B1 genotype). The statistical analysis was performed using GraphPad Prism (version 9, GraphPad Software, San Diego, CA, United States) and differences were considered statistically significant when p < 0.05. The phenotypic assessments were performed blinded to the genotype of the embryo, but not to the treatment group.

## Data Availability

The original contributions presented in the study are included in the article/Supplementary Material, further inquiries can be directed to the corresponding author.

## Supplementary material

SUPPLEMENTARY FIGURE 1 Female skew in NTD from chow and HFHS-fed females. Sex ratio in SR-B1 KO NTD embryos retrieved at E9.5 from dams fed chow or HFHS diet. The number of embryos is shown inside the bars.

SUPPLEMENTARY FIGURE 2 Distribution of developmentally delayed embryos among litters from control and HFHS groups. Number of somites/embryo in 10 representative litters from dams exposed to **(A)** control or **(B)** HFHS diet. **(C)** Mean number of developmentally delayed embryos per pregnancy for chow and HFHS groups (*p < 0.05, Student’s t-test).

SUPPLEMENTARY FIGURE 3 Lipoperoxidation estimated by TBARS levels in chow and HFHS-fed females at E9.5. **(A)** TBARS concentration in plasma (n = 5/group). **(B)** TBARS concentration in liver homogenates (n = 8/group).
