# Supplementary material for: Vitamin E supplementation prevents obesogenic diet-induced developmental abnormalities in SR-B1 deficient embryos
Source: Front Cell Dev Biol. 2024 Oct 9;12:1460697. doi: 10.3389/fcell.2024.1460697 (PMC11496146; doi:10.3389/fcell.2024.1460697)
Supplement: Supplementary file 1 [file Table1.docx]

**Supplementary Table I.**

|  | **CHOW** | **HF DIET** | **HF DIET + VITAMIN E** |
| --- | --- | --- | --- |
|  | **Prolab RMH3000** | **DIO Rodent Purified 58Y1** | **DIO Rodent Putified + vitamin E** |
| **Protein, %** | **22.5** | **22.6** | **23.1** |
| Arginine, %  Cystine, %  Glycine, %  Histidine, %  Isoleucine, %  Leucine, %  Lysine, %  Methionine, %  Phenylalanine, %  Tyrosine, %  Threonine, %  Tryptophan, %  Valine, %  Serine, %  Aspartic Acid, %  Glutamic Acid, %  Alanine, %  Proline, %  Taurine, % | 1.37  0.30  1.11  0.53  1.14  1.68  1.31  0.49  1.00  0.60  0.83  0.30  1.12  1.20  2.35  5.33  1.17  1.73  0.02 | 1.01  0.54  0.49  0.72  1.37  2.43  2.12  0.80  1.34  1.52  1.11  0.34  1.71  1.52  1.89  5.84  0.78  2.82  0.00 | 0.90  0.48  0.50  0.67  1.24  2.24  1.88  0.67  1.24  1.31  1.00  0.29  1.47  1.43  1.66  5.28  0.71  3.04  0.00 |
| **Fat %** | **5.4** | **35.1** | **34.9** |
| Cholesterol, ppm  Linoleic Acid, %  Linolenic Acid, %  Arachidonic Acid, %  Omega-3 Fatty Acids, %  Total Saturated FAs,  % Total MUFAs % | 195  1.73  0.16  0.00  0.34  1.75  1.60 | 301  4.70  0.39  0.06  0.39  13.79  14.09 | 301  4.70  0.39  0.06  0.39  13.68  14.00 |
| **Fiber %** | **4** | **6.5** | **6.5** |
| **Carbohydrates %** | **52** | **25.9** | **25.5** |
| Starch/maltodextrin % | 30 | 16.1 | 16.1 |
| Sucrose % | 1 | 8.8 | 8.8 |
| **Vitamins** |  |  |  |
| Carotene, ppm  Vitamin K ppm  Thiamin HCl, ppm  Riboflavin, ppm  Niacin, ppm  Pantothenic Acid, ppm  Choline Chloride, ppm  Folic Acid, ppm  Pyridoxine, ppm  Biotin, ppm  B12, mcg/kg  Vitamin A, IU/gm  Vitamin D3 IU/gm  Vitamin E, IU/kg  Ascorbic Acid, mg/gm | 2.6  1.9  10  14  63  13  1600  1.2  7.6  0.38  75  29  2.4  75  0 | 5.2  0.65  6.1  7.8  39  19  1290  2.6  7.4  0.3  12  5.2  1.3  67.2  0 | 5.2  0.65  6.1  7.8  39  19  1290  2.6  7.4  0.3  12  5.2  1.3  2005  0 |
| **Minerals** |  |  |  |
| Calcium, %  Phosphorus, %  Potassium, %  Magnesium, %  Sodium, %  Chlorine, %  Fluorine, ppm  Iron, ppm  Zinc, ppm  Manganese, ppm  Copper, ppm  Cobalt, ppm  Iodine, ppm  Chromium, ppm  Selenium, ppm | 1.00  0.75  0.91  0.24  0.26  0.44  16  380  120  96  12  0.27  0.98  1.4  0.21 | 0.79  0.56  0.77  0.07  0.15  0.24  1.2  62  46  76  8.1  0.0  0.27  2.6  0.21 | 0.79  0.56  0.77  0.07  0.15  0.24  1.2  62  46  76  8.1  0.0  0.27  2.6  0.21 |
| **CALORIES (kcal/gm)** | **3.46** | **5.10** | **5.08** |
| % calories from protein | 26 | 18 | 18 |
| % calories from fat | 14 | 62 | 62 |
| % calories from carbohydrates | 60 | 20 | 20 |
| **INGREDIENTS** |  |  |  |
|  | Ground wheat, soybean meal, wheat, corn, fish meal, porcine fat preserved with BHA, dehydrated alfalfa, calcium carbonate, brewers dried yeast, soybean oil, salt, dicalcium phosphate, monocalcium phosphate, salt, DL-methionine, L-lysine, choline chloride, vitamin A acetate, menadione dimethylpyrimidinol bisulfite, magnesium oxide, ferrous sulfate, pyridoxine hydrochloride, cholecalciferol, biotin, dl- alpha tocopheryl acetate, vitamin B12 supplement, riboflavin, thiamin mononitrate, zinc oxide, folic acid, calcium pantothenate, nicotinic acid, manganous oxide, ferrous carbonate, copper sulfate, zinc sulfate, calcium iodate, cobalt carbonate, sodium selenite. | Lard Casein - Vitamin Free, Maltodextrin, Sucrose, Powdered Cellulose, Soybean Oil,  Potassium Citrate, Dicalcium Phosphate,  DIO Mineral Mix, AIN-76A Vitamin Mix, Calcium Carbonate, L-Cystine, Choline Bitartrate, Blue Dye | Lard, Casein - Vitamin Tested,  Maltodextrin,  Sucrose,  Powdered Cellulose,  Soybean Oil,  Potassium Citrate, Calcium Phosphate,  AIN-76A Vitamin Mix for 2000 IU/kg vitamin E, DIO Mineral Mix  Calcium Carbonate  L-Cystine  Choline Bitartrate, Green Dye |
