# Supplementary figures and images for: Vitamin E supplementation prevents obesogenic diet-induced developmental abnormalities in SR-B1 deficient embryos
Source: Front Cell Dev Biol. 2024 Oct 9;12:1460697. doi: 10.3389/fcell.2024.1460697 (PMC11496146; doi:10.3389/fcell.2024.1460697)

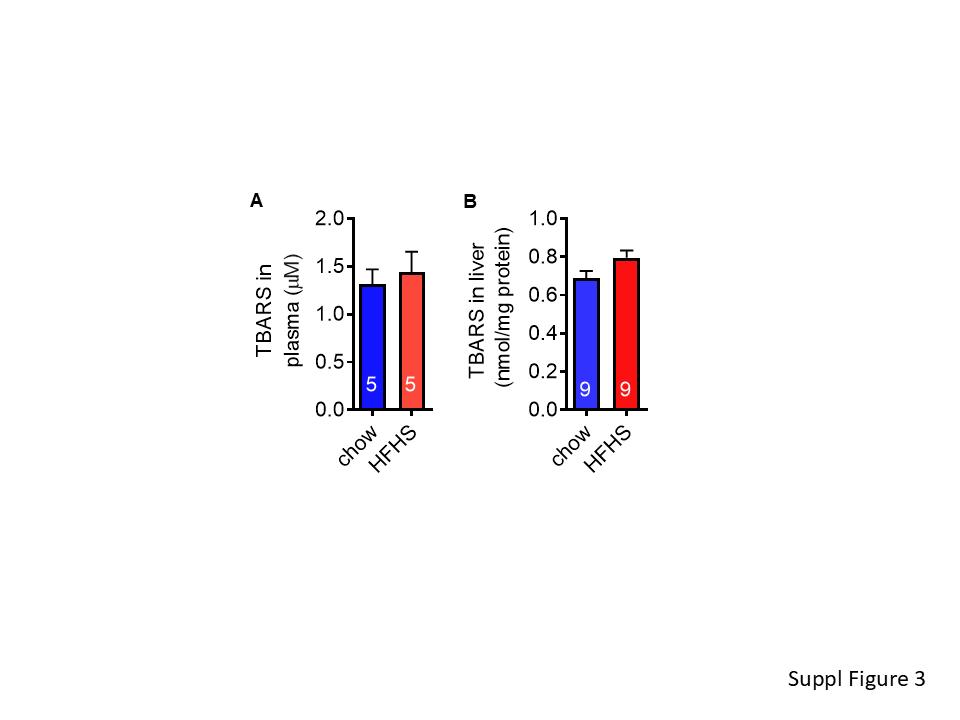

Supplement: Supplementary file 2 [file Image3.tif]

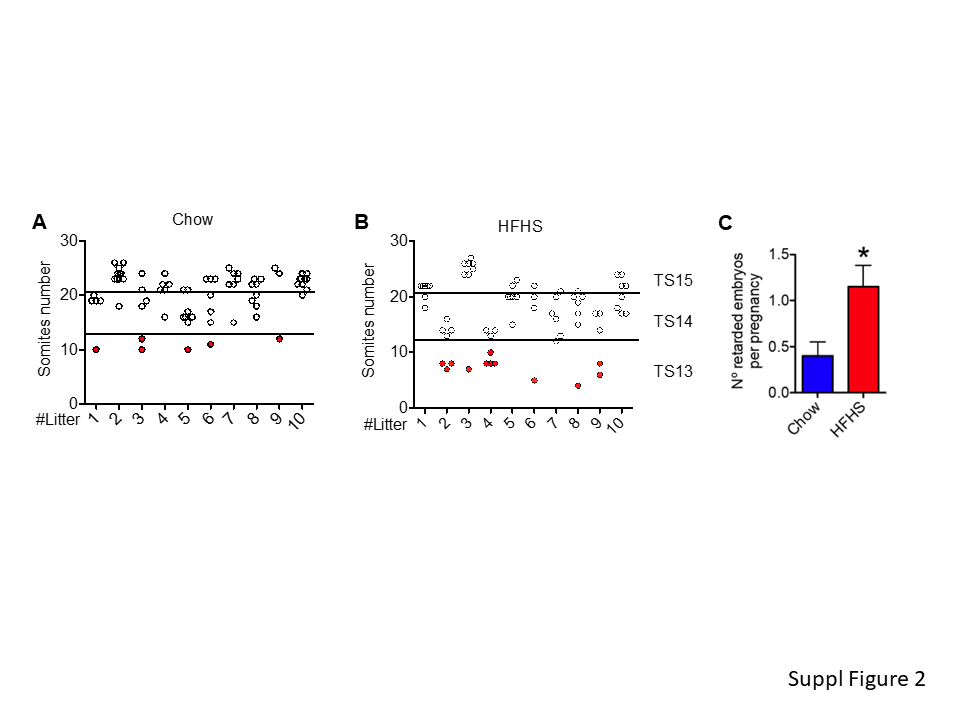

Supplement: Supplementary file 3 [file Image2.TIF]

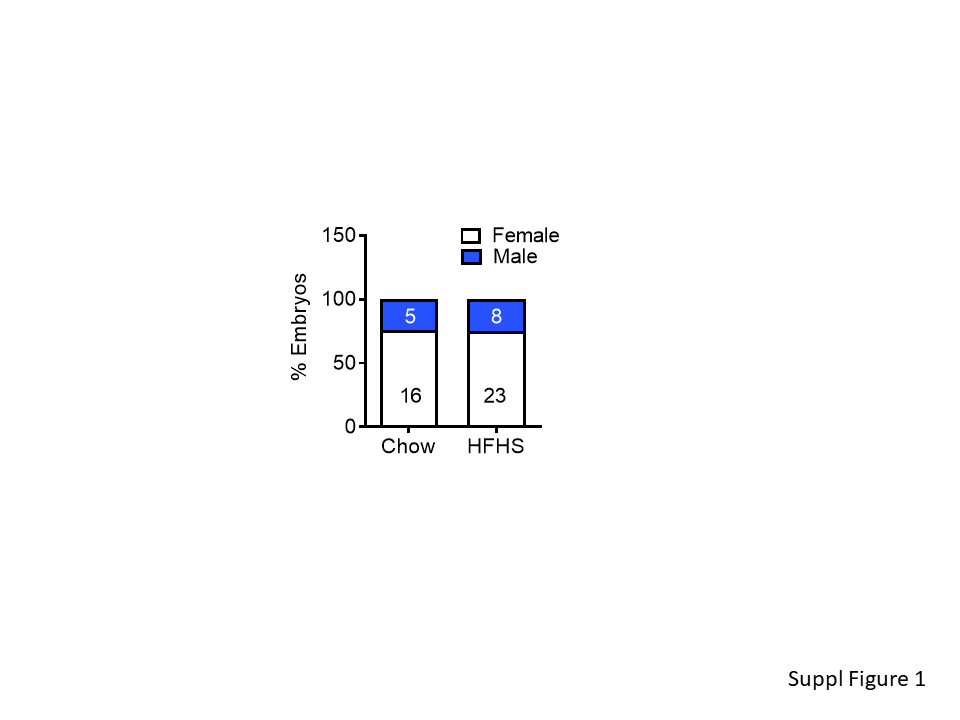

Supplement: Supplementary file 4 [file Image1.TIF]
